# Developing a complex intervention for diet and activity behaviour change in obese pregnant women (the UPBEAT trial); assessment of behavioural change and process evaluation in a pilot randomised controlled trial

**DOI:** 10.1186/1471-2393-13-148

**Published:** 2013-07-15

**Authors:** Lucilla Poston, Annette L Briley, Suzanne Barr, Ruth Bell, Helen Croker, Kirstie Coxon, Holly N Essex, Claire Hunt, Louise Hayes, Louise M Howard, Nina Khazaezadeh, Tarja Kinnunen, Scott M Nelson, Eugene Oteng-Ntim, Stephen C Robson, Naveed Sattar, Paul T Seed, Jane Wardle, Thomas AB Sanders, Jane Sandall

**Affiliations:** 1 Division of Women’s Health, Women’s Health Academic Centre, King’s College London and King’s Health Partners, 10th floor, North Wing, St.Thomas’ Hospital, London SE1 7EH, UK; 2 Division of Diabetes and Nutritional Sciences, King’s College London and King’s Health Partners, London, UK; 3 Institute of Health & Society, Newcastle University, Newcastle, UK; 4 Institute of Cellular Medicine, Newcastle University, Newcastle, UK; 5 Department of Health Sciences, University of York, Newcastle, UK; 6 Epidemiology and Public Health, University College London, London, UK; 7 School of Medicine, University of Glasgow, Glasgow, UK; 8 School of Health Sciences, University of Tampere, Tampere, Finland

**Keywords:** Pregnancy, Obesity, Diet, Physical activity, Complex intervention, Evaluation

## Abstract

**Background:**

Complex interventions in obese pregnant women should be theoretically based, feasible and shown to demonstrate anticipated behavioural change prior to inception of large randomised controlled trials (RCTs). The aim was to determine if a) a complex intervention in obese pregnant women leads to anticipated changes in diet and physical activity behaviours, and b) to refine the intervention protocol through process evaluation of intervention fidelity.

**Methods:**

We undertook a pilot RCT of a complex intervention in obese pregnant women, comparing routine antenatal care with an intervention to reduce dietary glycaemic load and saturated fat intake, and increase physical activity. Subjects included 183 obese pregnant women (mean BMI 36.3 kg/m^2^).

Diet was assessed by repeated triple pass 24-hour dietary recall and physical activity by accelerometry and questionnaire, at 16^+0^ to 18^+6^ and at 27^+0^ to 28^+6^ weeks’ gestation in women in control and intervention arms. Attitudes to behaviour change and quality of life were assessed and a process evaluation undertaken. The full RCT protocol was undertaken to assess feasibility.

**Results:**

Compared to women in the control arm, women in the intervention arm had a significant reduction in dietary glycaemic load (33 points, 95% CI −47 to −20), (p < 0.001) and saturated fat intake (−1.6% energy, 95% CI −2.8 to −0. 3) at 28 weeks’ gestation. Objectively measured physical activity did not change. Physical discomfort and sustained barriers to physical activity were common at 28 weeks’ gestation. Process evaluation identified barriers to recruitment, group attendance and compliance, leading to modification of intervention delivery.

**Conclusions:**

This pilot trial of a complex intervention in obese pregnant women suggests greater potential for change in dietary intake than for change in physical activity, and through process evaluation illustrates the considerable advantage of performing an exploratory trial of a complex intervention in obese pregnant women before undertaking a large RCT.

**Trial registration:**

Trial Registration Number: ISRCTN89971375

## Background

Obesity is prevalent in women of reproductive age in both high and low-to-middle income countries [1]. Pregnant obese women have a heightened risk of adverse pregnancy outcomes [2], but at present there is no evidence-based intervention that can be introduced into clinical practice to improve pregnancy outcome in obese women. The majority of attempts to develop interventions have hitherto focused on limiting gestational weight gain (GWG) according to the USA Institute of Medicine (IOM) recommendations [3]. Recent meta-analyses of relevant studies in obese women show modest restriction of GWG without robust evidence for improved clinical outcome [4,5]. Limitations of the existing evidence include poor study design, small sample size, absence of a theoretical basis and, importantly, no *a priori* demonstration of the feasibility of the intervention in regard to changing the specific behaviours targeted [6]. We have developed a theoretically based behavioural group intervention (diet and physical activity) for obese pregnant women with the primary aim of improving maternal glucose homeostasis. As maternal insulin resistance is integral to many complications of obese pregnancy the dietary intervention focuses on lowering the dietary glycaemic index (GI), previously shown to improve pregnancy outcome in women with gestational diabetes (GDM) [7,8]. Increased physical activity can also improve metabolic control and reduce GDM risk in pregnant women [9].

Prior to embarking on a large randomised controlled trial (RCT) and in accordance with UK Medical Research Council Guidance for development of a complex intervention [10], we first explored the theoretical basis for an intervention in obese pregnant women [5,6,11,12], leading to development of a novel intervention (Phase 1). We now report on Phase 2, an exploratory trial to determine whether this intervention achieved the changes in dietary and physical activity behaviours anticipated, and to undertake a process evaluation of every aspect of fidelity of the intervention and the protocol.

### Subjects

Potentially eligible participants attending clinics for general antenatal care were approached by research midwives in four UK study centres in urban settings providing a range of models of care. The contributing hospitals were 1) The Southern General Hospital and Princess Royal Maternity Hospital (Glasgow), 2) The Royal Victoria Infirmary (Newcastle), 3) Guy’s and St Thomas’ NHS Foundation Trust (London) and 4) King’s College Hospital Foundation Trust (London).

## Methods

**Figure 1:**
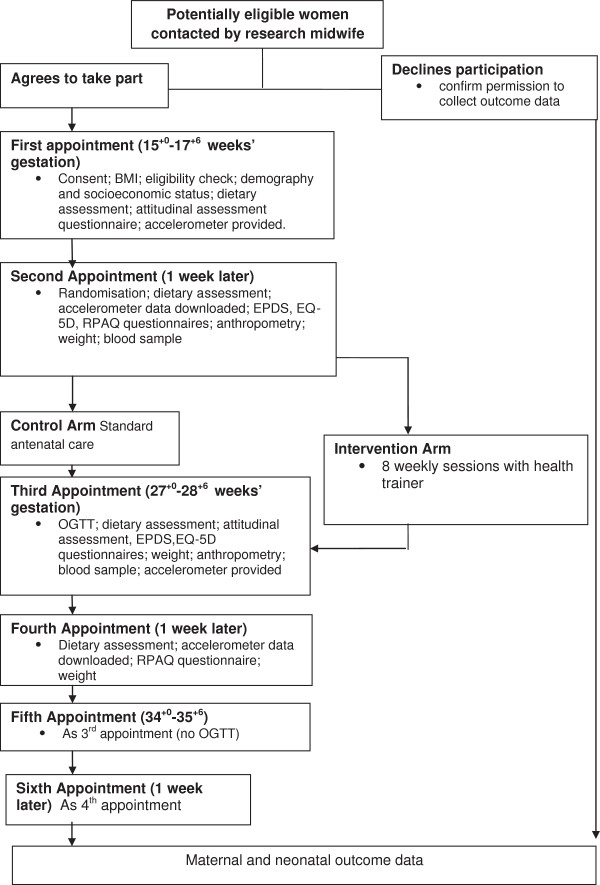
**Study protocol.** Abbreviations: BMI, Body Mass Index; EQ-5D, EuroQuol Quality of Life Questionnaire; EPDS, Edinburgh postnatal depression score questionnaire; OGTT, oral glucose tolerance test; RPAQ, Recent Physical Activity Questionnaire.

The protocol for the exploratory trial is shown in Figure 1.

Verbal and printed information was provided to potential participants at a routine antenatal appointment in the first trimester and women were contacted >24 hrs later to ascertain willingness to participate. For those declining participation, consent to record basic demographic data and BMI were obtained. Those willing to participate were invited to return for their first study appointment in the early second trimester (>15^+0^ weeks to <17^+6^ weeks’ gestation). This window of recruitment allowed adequate time for arrangement of the one to one session with the health trainer followed by the eight week intervention programme prior to the oral glucose tolerance test, carried out between 27^+0^ and 28^+6^ weeks’ gestation. Research midwives received a study-specific manual, attended at least one training session with the trial manager and continued feedback and training sessions for the study duration.

Inclusion criteria: BMI ≥30 kg/m^2^ and singleton pregnancy; gestational age >15^+0^ weeks and <17^+6^ weeks’ gestation.

Exclusion Criteria: Unable or unwilling to give written informed consent; gestation <15^+0^ weeks and >17^+6^ weeks; pre-existing diabetes; pre-existing essential hypertension (treated); pre-existing renal disease; multiple pregnancy; systemic lupus erythematosus (SLE); antiphospholipid syndrome; sickle cell disease; thalassemia; celiac disease; currently prescribed metformin; thyroid disease or current psychosis.

All data were entered onto a password protected secure database (MedSciNet Ltd). Randomisation was performed online. The randomised treatment was allocated automatically, balanced by minimisation for maternal age, centre, ethnicity, parity and BMI. Data were analysed using Stata (version 11.2, StataCorp, College Station, Texas). All women randomised between 29th March 2010 and 13th May 2011 were included. Postcodes were matched to two national indices of deprivation: the Index of Multiple Deprivation (IMD) for English addresses, or the Scottish Index of Multiple Deprivation (SIMD) for addresses in Scotland [13,14].

### Control arm; standard care

Following randomisation, women in the control arm returned for data collection appointments with the study midwife at 27^+0^ -28^+6^ and 34^+0^-36^+6^ weeks’, where possible coinciding with routine antenatal visits.

### Intervention arm

Following randomisation, participants attended a one-to-one appointment with the health trainer (HT) and were invited to weekly group sessions for 8 consecutive weeks from approximately 19 weeks’ gestation.

All women attended routine antenatal care appointments and received advice regarding diet and physical activity (PA) in accordance with local policies, which draw on UK NICE guidelines [15].

### Sample size

The primary outcome was change in dietary and PA behaviours at 28 weeks’ gestation (coinciding with the primary maternal outcome for the main RCT, GDM at 28 weeks’). No prior investigation in obese pregnant women was available to inform power at the planning stage. The sample size of 183 was determined by the predefined duration of Phase 2, the exploratory phase. This number was adequate to enable power calculations for primary endpoints of the subsequent RCT, by providing estimates of the variance to within approximately 7% of the true value.

### Ethics

Research Ethics Committee approval was obtained in all participating centres, UK Integrated Research Application System; reference 09/H0802/5 (South East London Research Ethics Committee).

### The intervention

The intervention was informed by psychological models of health behaviour including control theory [16] and social cognitive theory [17]. Although no clear patterns between intervention characteristics and outcomes have been seen to date in lifestyle interventions in pregnancy, and few studies have described their theoretical basis [5,6]. Self-regulation techniques, drawn from control theory, suggest that behaviour change is facilitated by feedback about performance compared to pre-specified goals [16,18]. This approach was utilised in this study by setting ‘SMART’ (Specific, Measurable, Achievable, Relevant, and Time Specific) diet and activity goals, with behaviours recorded in a log book. Identification of benefits and overcoming barriers to behaviour change, and increasing self-efficacy were also included, and social support facilitated through the group format [17]. Following initial feedback from HTs regarding difficulties encountered by some women in attending sessions, for those women unable to attend, the session content was delivered by phone or email.

### Dietary advice

Pre-specified dietary outcomes were a change in GI, glycaemic load (GL) (an indicator of carbohydrate quality (GI) and quantity consumed), and energy intake from saturated fatty acids (SFA). The focus of the dietary advice to the intervention group was therefore on increased consumption of foods with a low dietary GI, including replacement of sugar sweetened beverages with low GI alternatives. Reduction in saturated fats and replacement with monounsaturated and polyunsaturated fat was also recommended. Exchange of foods was emphasised e.g. a high GI food for a low GI food, rather than limiting energy intake.

### Physical activity advice

Women in the intervention arm were encouraged to increase daily PA incrementally, setting goals of incremental step counts (monitored by pedometer) and maintaining the achieved PA level after the intervention period. Recommendations included an emphasis on walking at a moderate intensity level [19].

### Intervention delivery

The intervention was delivered by health trainers (HTs). In the UK, HTs do not have pre-specified health professional qualifications, but relevant experience (http://informationstrategy.dh.gov.uk/health-trainer-workforce). All HTs received a comprehensive treatment manual, pre-study training (and within-study supervision) in behaviour modification and conducting group sessions (organised by Weight Concern; Registered Charity 1059686). The sessions were held in a hospital setting in all but one centre, where women attended a community children’s centre. At the initial one-to-one appointment women were provided with a participant handbook, reflecting the rationale and content of the HT sessions, a pedometer (Yamax SW-200 Digiwalker), a log book for weekly SMART goals and related behaviours (steps, PA and diet) and a DVD of a specially devised pregnancy exercise regime. Potential benefits of attending group sessions were discussed. Each group session delivered a different element of the dietary and PA intervention. Additional file 1: Table S1. Goals from the previous week were reviewed and goals set for the following week. Discussion included barriers to behavioural change and ways these might be overcome.

The following information was obtained from all participants (at visits indicated in Figure 1).

### Attitudinal assessment questionnaire

The attitudinal assessment included questions relating to perceived benefits and barriers and confidence to carry out the dietary and PA behaviours [20,21]. The target behaviours were to consume lower GI carbohydrates, to reduce saturated fat intake and to increase PA.

### Health status and mental health

The EuroQol quality of life (EQ- 5D) questionnaire [22] was used to assess health status, and the Edinburgh Post Natal Depression Score (EPDS) to assess mental health [23].

### Dietary assessment

Repeated, triple pass 24 hr recall data obtained at baseline (randomisation) and 28 weeks’ gestation were evaluated twice, one week apart in both the intervention and control group. The 24 hr dietary recall is a standard retrospective, interviewer led dietary assessment methodology used to capture information on all food and drinks consumed in the preceding 24 hrs. This is carried out in three stages (the triple pass), which includes 1) recording a ‘quick’ list of foods eaten or drunk, 2) collecting more detailed information of these foods and 3) reviewing all items once more in order to clarify any ambiguities or omissions. A short food frequency questionnaire (FFQ), for later validation, was also completed.

### Physical activity assessment

At the first and third appointments participants were asked to wear an Actigraph™ accelerometer (Florida, USA) (either GT1M or GT3X set to uniaxial mode) for seven consecutive days, removing it for washing, bathing, swimming and at night. PA was also assessed by questionnaire (Recent Physical Activity Questionnaire (RPAQ).

### Process evaluation

A process evaluation, following Steckler and Linnan’s [24] framework was undertaken. This explored 1) Context (environmental, socio-economic or political factors), 2) Reach (the proportion of the intended target audience that participates, and which subgroups, if any, do not participate), 3) Dose delivered and dose received (the proportion of intended intervention received) 4) Fidelity (if each component of the complex intervention was provided as intended) and 5) Acceptability (if the intervention materials and advice were well received by providers and participants).

Qualitative semi-structured interviews were conducted, to capture women’s experiences and perceptions of the trial and intervention. Women were recruited from each of the participating study sites using a maximum diversity sampling approach, following an informed consent procedure. Interviews took place between November 2010 and February 2011, and were either face-to-face (n = 17), mostly in hospital settings, or by phone (n = 4). Control (n = 12) and intervention (n = 9) interviewees were asked about their involvement in the research and their experiences of the trial appointments, measurements, blood tests and accelerometry recordings. Women in the intervention arm were additionally asked about their perceptions of the different components of the intervention, and how these impacted upon their lives. The interviews were conducted by one researcher and took place during pregnancy after the intervention had been provided. In addition, health trainers completed audio diaries (130 recordings) in which they reflected on the fidelity and feasibility of the intervention delivery. Attendance at sessions was recorded on the study database.

### Clinical outcome data

#### Maternal primary outcome for the subsequent RCT (diagnosis of GDM)

A blood sample for fasting glucose and insulin was taken after an overnight fast. For the OGTT, following a glucose load (410 ml of lucozade or 75 g glucose in water), 1 hr and 2 hr samples were taken for glucose measurement. Diagnosis of GDM was confirmed by fasting glucose ≥ 5.1 mmol/L and/or 1 hr glucose ≥10 mmol/L; 2 hr glucose ≥8.5 mmol/L according to the International Association of the Diabetes and Pregnancy Study Groups (IADPSG) guidelines [25]. Following GDM diagnosis, women were referred for routine GDM care according to local criteria.

#### Neonatal primary outcome for the subsequent RCT (large for gestational age delivery (LGA) defined as >90^th^ customised birthweight centile)

Customised birthweight centiles were calculated correcting for gestational age, maternal ethnicity, weight and height in early pregnancy, parity and infant sex [26]. Weight adjustment for women with BMI ≥30 kg/m^2^ is based on a notional weight corresponding to a BMI of 29.9 kg/m^2^.

### Outcome data also recorded (not reported)

These included maternal outcomes: diagnosis of GDM and pre-eclampsia, gestational weight gain, mode of delivery, blood loss at delivery, inpatient nights, detailed clinical and family history, health in current pregnancy, early pregnancy data (ultrasound scan, nuchal screening), blood pressure, routine blood results; neonatal outcomes: gestational age at delivery, birthweight, anthropometry, inpatient nights. Maternal urine and cord blood samples were also provided.

### Data handling and statistical analysis

#### Health quality and attitudinal assessment questionnaires

The generic EQ-5D health-related quality of life instrument [22] is reported as the proportion of women with problems on individual dimensions (mobility, self-care, usual activities, pain/discomfort, and anxiety/depression). It is given as a summary index score calculated from preference values of different combinations of the dimensions elicited using the time trade-off method in a sample representative of non-institutionalized adults in England, Scotland and Wales (range −0.59 to 100 where −0.59 is severe problems on all dimensions) [27,28]; and also the visual analogue scale (VAS) of health-related quality of life (range 0 to 100 where 0 is worst imaginable health state). The change between baseline and 28 weeks’ gestation in the percentage of women with any problem was assessed using McNemar’s test of changes. Attitudes to target behaviours (attitudinal assessment questionnaire) are based on the average of multiple responses on 5-point scales (3 responses for diet, 13 for PA) with 5 indicating the greatest perceived barrier, perceived benefit, or level of confidence.

#### Assessment of deprivation

These scales for estimation of deprivation in England and Scotland [13,14] use different reference populations to determine the actual indices of deprivation, and are therefore not directly comparable. For the purposes of this study, the most deprived quintile is presented separately for women in each population and compared to the remainder of the population (quintiles 1–4).

#### Dietary analysis

Quality of dietary data was checked within one week of entry. Dietary coding utilised McCance and Widdowson “Composition of Foods” (6^th^ edition) food codes and nutrient composition was evaluated using WISP 3.0 (Tinuviel Software) for GI and GL values [29]. Estimates using previously published methodology were made when GI values were not available [30]. 24 hr recall data obtained at baseline (randomisation) and at 28 weeks’ gestation was evaluated twice, one week apart, and data were averaged. The validity of the short FFQ was assessed against the dietary recall data. Pre-specified dietary outcomes were a change in GI, GL and energy intake from SFA. Total energy intake, the proportion of energy derived from macronutrients were assessed.

#### Actigraph analysis

An epoch length (time sampling interval) of 15 seconds was specified. Data were processed using the MAHUFFE Software package [31]. Sedentary behaviour was defined as <100 counts per minute (cpm), light activity as 100–1951 cpm, moderate intensity activity as 1952–5725 cpm and vigorous activity as > 5725 cpm [32]. As time spent in vigorous activity was very low, minutes of moderate and vigorous physical activity (MVPA) were combined. Runs of zero counts lasting >60 minutes were excluded, as these indicated monitor removal. A valid recording was defined as a day in which >500 minutes of monitored on-time were recorded in 24 hrs [33]. Data from participants recording ≥3 days of valid accelerometry data were included in the analysis. The specified PA outcome was an increase in minutes per day of MVPA recorded by accelerometry.

#### Recent physical activity questionnaire (RPAQ)

The RPAQ was modified for the assessment of PA in the preceding seven days. Estimates of minutes per day spent in light, moderate and vigorous activity in each of the domains were calculated. Sedentary activities were defined as those with a metabolic equivalent (MET) of <1.5. Light activities were those of 1.5 to 3 METs. Moderate activities were those of 3 to 6 METs. Vigorous activities were those of 6 METs or greater [34] MVPA were combined to give one summary variable.

#### Process evaluation

Interviews were recorded and transcribed verbatim. Transcripts were anonymised and a unique identifier (ID number) was used to maintain confidentiality. Data were imported into a qualitative software analysis package (NVivo 8), and subject to comparative thematic analysis [35]. To enhance study validity and reliability, themes arising from the research were discussed, the data supporting these was reviewed by co-researchers, and data were compared between sites and with existing literature. By these methods, assumptions were tested and observations of differences and their relationship to the theoretical models underpinning the study were explored.

#### Statistical analysis

Analyses followed the intention-to-treat principle. Following CONSORT guidelines, risk ratios and risk differences were estimated by binary regression for Yes/No outcomes. Where measures were repeated at baseline and 28 weeks’ gestation, results [mean (SD) n (%)] are presented separately at each time point. Randomised comparisons at 28 weeks’ were made using linear regression with robust standard errors, adjusting for the baseline value. For PA data, dummy variables were used when the baseline values were missing. Correlations between PA as assessed objectively (accelerometry) and when self-reported (RPAQ) were explored.

## Results

**Figure 2:**
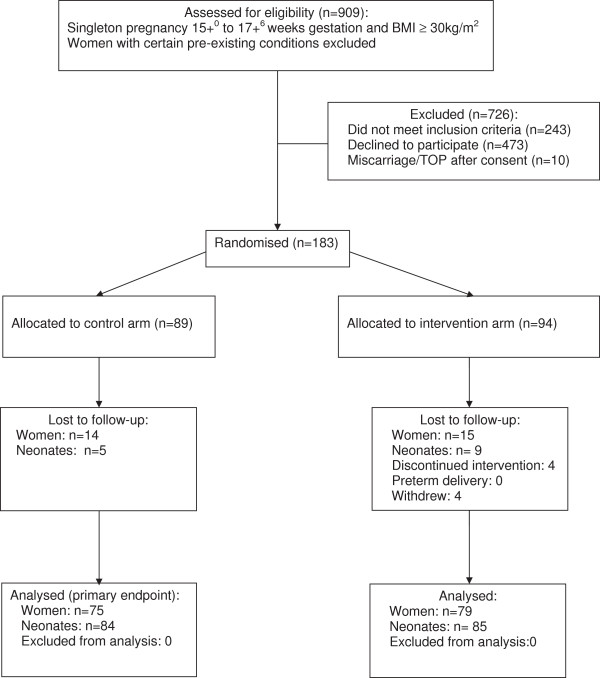
**Consort Diagram.** Flow chart of participants through study.

Figure 2 provides a flow chart of participants through the study.

### Participants

Mean first visit BMI was 36.3 kg/m^2^. More than half the women were White and the remainder from Black (38%) and Minority Ethnic communities. More than half (56%) already had at least one child. More than half of those from centres in England and over 40% in Scotland came from regions in the highest quintile of social deprivation (Table 1).

**Table 1:** **Description of subjects at baseline (16**^**+0**^**-18**^**+6**^ **weeks gestation) by randomised treatment**

	***Control***	***Intervention***
	***n = 89***	***n = 94***
Age (years) ^1^	30.7 (4.9)	30.4 (5.7)
*Age categories*		
18-25	16 (18%)	22 (23%)
26-30	25 (28%)	27 (29%)
31-40	46 (52%)	42 (45%)
41 plus	2 (2%)	3 (3%)
*Anthropometry*		
Height (m)	1.64 (0.07)	1.64 (0.07)
Weight (kg)	96.8 (16.2)	97.8 (12.7)
BMI (kg/m^2^)	36.1 (4.8)	36.5 (4.7)
*Ethnicity*^1^		
White	51 (57%)	52 (55%)
Black	32 (36%)	38 (40%)
Asian	1 (1%)	2 (2%)
Other	5 (6%)	2 (2%)
*Parity*^1^		
0	38 (43%)	42 (45%)
1	36 (40%)	29 (31%)
2 or more	15 (17%)	23 (24%)
*Cigarette smoking*		
Never	61 (68%)	63 (67%)
Ex-smoker	22 (25%)	25 (27%)
Current	6 (7%)	6 (6%)
*Number of cigarettes*		
0	83 (93%)	83 (88%)
1-5 per day	3 (3%)	3 (3%)
11-20 per day	1 (1%)	6 (6%)
6-10 per day	2 (2%)	2 (2%)
*Index of multiple deprivation*^2^		
England	n = 76	n = 79
Mean (SD)	34 (12)	36 (14)
Quintiles		
1-4 (less deprived)	35 (46%)	29 (37%)
5 (most deprived)	41 (54%)	50 (63%)
Scotland	n = 12	n = 14
Mean (SD)	28 (11)	30 (20)
Quintiles		
1-4 (less deprived)	7 (58%)	8 (57%)
5 (most deprived)	5 (42%)	6 (43%)
*Living arrangements*		
Single	35 (39%)	50 (53%)
With partner	66 (74%)	69 (73%)
With parent(s)	7 (8%)	13 (14%)
Without partner or parents	17 (19%)	17 (18%)
*Accommodation*		
Owned	27 (30%)	21 (22%)
Rented (private)	26 (29%)	27 (29%)
Rented (council)	36 (40%)	46 (49%)

Results shown are mean (SD) or n (%).

^1^ The randomised treatment allocation is balanced by minimization on maternal age, centre, ethnicity, and parity.

^2^ The index of multiple deprivation is calculated for the region of residence (Lower super output area in England, data region in Scotland) [13,14]. Different methods and reference populations are used in England and Scotland, and the indices are not directly comparable.

### Diet

Table 2 shows the dietary intakes at baseline and at 28 weeks of gestation. There were no differences between groups in energy intakes, GI, GL, or other macronutrient at baseline. However, following the intervention, at 28 weeks’ gestation, total energy intake, dietary GL, GL (%E), saturated fat (%E) and total fat (%E), were significantly lower and fibre intake measured as non-starch polysaccharides was greater in the intervention group than in the control arm. The proportion of energy derived from protein was higher in the intervention group but absolute protein intake did not differ. There was a difference of 7 GI points between the intervention and control group which achieved borderline statistical significance (P = 0.054).

**Table 2:** Dietary outcomes

		***Control***	***Intervention***	***Difference(95%CI)***	***P value***
n: baseline, 28 weeks’		n = 89, 69	n = 94, 71		
Total Energy (MJ/d)	Baseline	7.53 (2.21)	7.26 (2.29)		
	28 weeks	7.71 (2.30)	6.75 (2.57)	−0.94 (−1.72 to −0.18)	0.016
Dietary GI (%)	Baseline	58 (6)	58 (5)		
	28 weeks	60 (26)	53 (13)	−7 (−15 to 0)	0.054
Dietary GL (g/d)	Baseline	133 (48)	129 (41)		
	28 weeks	146 (55)	111 (39)	−33 (−47 to −20)	<0.001
GL (%E)	Baseline	27.7 (5.3)	28.5 (5.9)		
	28 weeks	31.3 (13.3)	26.6 (8.0)	−4.8 (−8.5 to −1.0)	0.013
Carbohydrate (%E)	Baseline	48.0 (8.4)	48.9 (9.6)		
	28 weeks	48.2 (8.0)	50.0 (8.2)	1.7 (−1.0 to 4.4)	0.207
Protein (%E)	Baseline	15.5 (3.6)	16.0 (4.2)		
	28 weeks	15.5 (3.2)	17.1 (4.9)	1.5 ( 0.1 to 2.8)	0.034
Protein (g)	Baseline	69.3(25.3)	68.5 (26.1)		
	28 weeks	70.6 (24.0)	66.5 (23.5)	−4.8 (−12.3 to 2.6)	0.204
Total fat (%E)	Baseline	36.0 (8.2)	34.9 (9.3)		
	28 weeks	35.9 (7.7)	32.5 (7.4)	−3.2 (−5.6 to −0.8)	0.010
SFA (%E)	Baseline	12.7 (3.9)	12.0 (4.3)		
	28 weeks	12.9 (3.9)	11.1 (3.8)	−1.6 (−2.8 to −0.3)	0.015
MUFA (%E)	Baseline	12.1 (4.1)	11.4 (4.0)		
	28 weeks	11.6 (4.0)	10.4 (3.2)	−1.0 (−2.2 to 0.2)	0.088
PUFA (%E)	Baseline	6.4 (3.0)	6.0 (3.1)		
	28 weeks	5.9 (2.8)	6.0 (2.7)	0.13 (−0.8 to 1.1)	0.774
P:S ratio	Baseline	0.56 (0.31)	0.56 (0.40)		
	28 weeks	0.51 (0.35)	0.64 (0.52)	0.13 (−0.01 to 0.28)	0.075
NSP (g)	Baseline	11.2 (4.6)	10.4 (4.6)		
	28 weeks	10.5 (4.2)	12.0 (6.0)	1.77 ( 0.08 to 3.47)	0.040

Abbreviations: *GI* glycaemic index, *GL* glycaemic load, *MUFA* monounsaturated fatty acid, *NSP* non-starch polysaccharide, *P:S ratio* polyunsaturated fatty acid, saturated fatty acid ratio; *PUFA* polyunsaturated fatty acid, *SFA* saturated fatty acids,%E: percentage energy.

For each dietary variable, results are presented in two lines: at trial entry (Baseline; 16^+0^-18^+6^ weeks’ gestation) and after randomised treatment, with comparisons and p-values only for the randomised comparison. Comparisons are adjusted for baseline levels throughout.

### Physical activity

There were no differences between the intervention and control arms in objectively measured PA variables at baseline or at 28 weeks’ gestation, after adjustment for baseline activity. Self-reported moderate to vigorous PA (MVPA) at 28 weeks’ gestation was increased in the intervention group (mean difference 34 minutes/day; 95% CI 9 to 59 min/day), but this was not supported by the objective data. Women in the intervention group self-reported walking for leisure for 14 min/day more than those in the control group at 28 weeks’ gestation (95% CI 5 to 23 min, p = 0.003). Agreement between the RPAQ questionnaire and accelerometry was very poor, for example, correlations between MVPA in the two formats at baseline were r = 0.275 (95% CI: 0.107 to 0.428) and at 28 weeks, r = −0.069 (95% CI: -0.296 to 0.165) Table 3.

**Table 3:** Physical activity as measured by accelerometer and RPAQ questionnaire

		***Control***	***Intervention***	***Treatment effect***
**By Accelerometer** n: baseline, 28 weeks		n = 72, 39	n = 68, 36	
Sedentary	Baseline	1172 (95)	1165 (91)	
	28 weeks	1175 (86)	1197 (77)	21 (−13 to 55)
Active	Baseline	217 (65)	225 (58)	
	28 weeks	209 (82)	194 (68)	−11 (−42 to 19)
Light	Baseline	178 (54)	184 (50)	
	28 weeks	175 (81)	161 (61)	−9 (−38 to 19)
MVPA	Baseline	40 (20)	42 (20)	
	28 weeks	34 (18)	33 (15)	−2 (−9 to 5)
**By RPAQ Questionnaire** n: baseline, 28 weeks		n = 80, 54	n = 79, 56	
Sedentary	Baseline	1007(207)	1009 (187)	
	28 weeks	1068(177)	1020 (226)	−50 (−115 to 16)
Active	Baseline	408 (189)	415 (180)	
	28 weeks	367 (175	410 (219)	45 (−16 to 106)
Light	Baseline	354 (180)	356 (164)	
	28 weeks	333 (165)	340 (204)	11 (−46 to 68)
MVPA	Baseline	54 (87)	60 (99)	
	28 weeks	34 (52)	70 (78)	34 (9 to 59)

Abbreviations: MVPA, Moderate and/or vigorous physical activity; RPAQ, Recent Physical Activity Questionnaire.

Results are measured in minutes/day presented as mean (SD). Treatment effects are mean differences (95% confidence intervals), adjusted for baseline activity; with dummy variables where baseline levels missing (2 accelerometer, 3 RPAQ).

### Attitudinal assessment of target behaviours

Benefits, barriers and confidence in making the target physical activity and dietary changes were unchanged in either the control and intervention groups from baseline to 28 weeks gestation Table 4.

**Table 4:** Attitudes to target behaviours, quality of life and mental health assessment

***Attitudes to target behaviours***		***Control***	***Intervention***	***Treatment effect***
***Barriers***				
Diet	Baseline	2.49 (0.58)	2.37 (0.61)	
	28 weeks	2.45 (0.58)	2.14 (0.68)	−0.18 (−0.35 to 0.00)
Physical activity	Baseline	2.64 (0.55)	2.48 (0.63)	
	28 weeks	2.47 (0.50)	2.20 (0.61)	−0.20 (−0.37 to −0.03)
***Perceived benefits***				
Diet	Baseline	3.75 (0.72)	3.80 (0.64)	
	28 weeks	3.79 (0.67)	3.97 (0.80)	0.13 (−0.10 to 0.36)
Physical activity	Baseline	3.94 (0.70)	4.04 (0.54)	
	28 weeks	3.84 (0.60)	4.06 (0.69)	0.17 (−0.04 to 0.38)
*Confidence*				
Diet	Baseline	3.78 (0.75)	3.84 (0.64)	
	28 weeks	3.71 (0.72)	3.85 (0.81)	0.11 (−0.15 to 0.37)
Physical activity	Baseline	3.76 (0.88)	3.92 (0.81)	
	28 weeks	3.77 (0.88)	3.81 (1.06)	−0.05 (−0.40 to 0.30)
***Quality of life (EQ-5D)***		***Control***	***Intervention***	***Treatment effect***
n: baseline, 28 weeks		n = 87, 75	n = 94, 80	
***Numbers reporting problems***				
Mobility	Baseline	10 (11%)	11 (12%)	
	28 weeks	21 (28%)	25 (31%)	4% (−10 to 18)
*Change (all women)*				*19*% *(11 to 27)*
Self-care	Baseline	1 (1%)	0 (0%)	
	28 weeks	3 (4%)	3 (4%)	−0.3% (−6 to 6)
*Change (all women)*				*4*% *(0 to 8)*
Usual activities	Baseline	16 (18%)	13 (14%)	
	28 weeks	26 (34%)	26 (33%)	−1% (−15 to 12)
*Change (all women)*				*16*% *( 8 to 24)*
Pain & discomfort	Baseline	38 (43%)	34 (36 %)	
	28 weeks	45 (60%)	54 (67%)	10% (−1 to 22)
*Change (all women)*				*25*% *(17 to 34)*
Anxiety & depression	Baseline	22 (25%)	20 (21%)	
	28 weeks	11 (15%)	17 (21%)	5% (−4 to 15)
*Change (all women)*				*−6*% *(−14 to 1)*
TTO score	Baseline	0.85 (0.18)	0.88 (0.14)	
	28 weeks	0.79 (0.24)	0.79 (0.16)	−0.03 (−0.07 to 0.02)
*Change (all women)*				*−0.08 (−0.10 to −0.06)*
VAS (0 to 100)	Baseline	76 (20)	76 (21)	
	28 weeks	75 (21)	78 (21)	4 (−3 to 10)
*Change (all women)*				*−2 (−6 to 2)*
***EPDS***				
Total	Baseline	7.1 (4.6)	7.4 (4.5)	
	28 weeks	6.9 (4.2)	7.1 (5.2)	0.1 (−1.1 to 1.3)
Total Score > 9	Baseline	25 (29%)	28 (30%)	
	28 weeks	17 (23%)	21 (26%)	1% (−9 to 11)
Total score > 12	Baseline	9 (10%)	10 (11%)	
	28 weeks	6 (8%)	14 (18%)	7% (−1% to 16)

Attitude to target behaviours are based on the average of multiple responses on 5-point scales, with 5 indicating the greatest barrier, perceived benefit, or level of confidence, and 1 the least.

Abbreviations: *EQ-5D* EuroQol 5 dimensional quality of life scale, *TTO* Time Trade Off health state ratings calculated from standard values elicited using the time trade-off method. *VAS* visual analogue scale, *EPDS* Edinburgh Postnatal Depression Score. Summaries are n (%) or mean (SD) as appropriate. For the EuroQoL subscales, the overall change over time is estimated as a risk difference, by McNemar’s test of changes. Elsewhere, differences are calculated by linear or binomal regression as appropriate, adjusting for baseline values.

### Health Status and Mental Health EPDS EQ-5D

There was no influence of the intervention on the numbers of women reporting problems in each of the EQ-5D domains, but as a group, obese women experienced a significant increase in problems with mobility, self care, usual activities and pain and discomfort from baseline to 28 weeks’ gestation. There was a 10% prevalence of probable depression at baseline and 13% at 28 weeks (i.e. EPDS score>12) with no significant effect of the intervention on anxiety and depression at 28 weeks Table 4.

### Process evaluation

#### Context

This study coincided with publication of new reports and guidance for obesity in pregnancy with associated media coverage [15,36]. Most control group interviewees demonstrated awareness and reported taking steps to improve their diet or fitness. Additional file 1: Table S2.

#### Reach

Those approached who were eligible for recruitment but declined to participate (n = 473) were of mean age 29.9 years; mean BMI 35.39 kg/m^2^; ethnicity, 59.7% White, 32.8% Black and 43.0% were in the lowest quintile for Index of deprivation indicating the most severe deprivation. Characteristics of participants providing semi-structured interviews (n = 21) are shown in Additional file 1: Table S3. This demographic profile was similar to study participants (Table 1). Overall, 29/183 (15.8%) women were lost to follow up Figure 2.

#### Dose

Of the 94 women randomised to the intervention, 82 (88%) attended at least one group session, and 60 (64%) attended 4 or more. A total of 42 women (45%) received material from all eight sessions, 6 by full attendance (6%) and the remainder when partly/wholly covered by subsequent phone contact. For all women, 6.1 (SD 2.6) sessions were attended or partly/wholly covered.

#### Fidelity

The intervention package (8 HT group sessions) was provided with good consistency at each study site. Goals were set at all group sessions, of which 88% were considered SMART by HTs according to their diaries. The maximum group size was 5 (mean 2).

#### Acceptability

Women in both arms of the trial found the research processes acceptable, and felt supported by the study midwives. Women in the intervention group were generally willing, in principle, to attend the eight health trainer sessions, and most women who attended valued the group approach, citing opportunities to raise questions and discuss each other’s experiences. Some were surprised at the extent of the intervention, having anticipated a less intensive, more advice-based approach.

Consistency of attendance at the HT sessions varied for different reasons including work commitments, school pick-up times, or feeling too unwell or tired. Occasionally initial involvement waned when groups proved smaller than anticipated, although the HT input by phone or email was considered valuable.

Some women found the information contained in the handbook new, whilst for others it was too basic. The pedometers and step goals were generally well received. Setting and reflecting on weekly goals was motivational for most, but could also invoke feelings of guilt, or a sense of being observed and judged. Women reported having watched the DVD, but few used it regularly. When interviewees were asked whether they had made any changes as a result of the intervention, most reported some degree of change, especially in relation to dietary intake. Reported changes in PA were more limited, particularly due to pelvic pain or tiredness as pregnancy progressed. Women often reported aspirations to increase exercise postnatally. See Additional file 1: Table S3 and S4 for extracts of interviews.

#### Maternal and neonatal outcomes

The primary maternal and neonatal outcomes for the subsequent RCT are shown in Table 5. There were no significant differences in GDM or LGA (≥90^th^ customised centile) between control and intervention arms. There was also no significant difference in gestational weight gain between control and intervention arms (secondary outcome).

**Table 5:** Maternal and neonatal primary outcomes

	***Control***	***Intervention***	***Comparison***	***Treatment effect***	***P value***
**Maternal**	n = 75	n = 79		(95% CI)	
GDM	24 (32%)	22 (28%)	Risk difference	−4% (−19 to 13)	0.574
			Risk ratio	0.87 (0.54 to 1.41)	
**Neonatal**	n = 84	n = 86			
LGA	7 (8%)	7 (8%)	Risk difference	0% (−8 to 8)	0.982
			Risk ratio	0.99 (0.36 to 2.7)	
>4 Kg	16 (19%)	13 (15%)	Risk difference	−4% (−15 to 8)	0.518

Abbreviations: *GDM* Gestational Diabetes Mellitus by IADPSG criteria, *LGA* Large for Gestational Age Delivery defined as ≥ 90th customized birthweight centile [26]. Customized centiles are adjusted for maternal age, height, weight, ethnic group, gestational age and gender. Weight adjustment for women with BMI ≥ 30 Kg/m^2^ (all women in study) is based on a notional weight corresponding to a BMI of 29.9 Kg/m^2^.

Continuous variables are given as mean (SD) with Mean Difference & 950025 CI. Binary outcomes are n (%), with Risk Difference and risk ratio.

The overall incidence of GDM, the primary outcome of the subsequent RCT (not powered for), according to recent International Association of the Diabetes and Pregnancy Study Groups (IADPSG) criteria [25] was 30%, enabling calculation of the subsequent RCT sample size (1546 women) for the RCT, powered for a 25% reduction. Since 38% of potentially eligible women took part in the pilot study, to achieve this sample size in the main RCT, approximately 4100 would need to be approached.

## Discussion

This study describes a pragmatic and rigorously evaluated pilot study of a complex intervention for diet and activity behaviour change in obese pregnant women. The intervention was associated with a significant change in dietary behaviour. Process evaluation showed overall acceptability of the protocol but led to several refinements to improve acceptability and fidelity.

In any lifestyle intervention of diet and physical activity, it is important that the pilot study design includes methods to assess the potential of the intervention to change these behaviours in the anticipated direction of effect. Few assessments of dietary intake in similar investigations of overweight or obese women have been attempted [37-39]. We used a 24 hr recall method to assess dietary intake and while this may lead to under-reporting of energy intakes, the reported values are not dissimilar to those of non-pregnant women in general UK population [40]. The objectives of the dietary intervention, to bring about reductions in GL and the proportion of energy derived from saturated fatty acids, were both achieved. This suggests that obese pregnant women are amenable to changing their diet in response to an intervention based on established theory, and that dietary advice, frequently delivered by health professionals, is likely to be successful in achieving dietary change in obese pregnant women, as previously implied [38]. The reduction in dietary GL achieved was similar (33% v 45%) to that reported in obese type 2 diabetic non-pregnant subjects in which improved glycaemic control was achieved [41]. Recently a similar intervention in 759 pregnant women, showed a lower change in GL (13%), which was associated with a reduction in gestational weight gain in women who had previously delivered a large for gestational age infant [42].

The reduction in energy intake observed is consistent with other studies that have restricted the intake of fat from meat and dairy products which have not been replaced by other sources of food energy [43]. The reduced GL may also have contributed through effects on satiety [44]. To our knowledge this is the first study demonstrating that anticipated changes in diet occur following delivery of an intervention to lower GL and saturated fat in obese pregnant women without GDM. Importantly this occurred despite the focus being on reducing GL by lowering the intake of added sugars as well as advocating foods with a lower GI. Focusing on GI tends to modify the GL from starch whereas the GI from sugar sweetened beverages is less amenable to change. Consequently, dietary advice to decrease the intake of added sugar, particularly as sugar-sweetened beverages, is likely to have had an important impact on GL.

This study adds to the scant literature on the habitual diet of obese pregnant women. The macronutrient profile at randomisation was similar to that of women in the general population, with fibre (non-starch polysaccharide) intake below, and total sugars and saturated fat above recommended UK guidelines [45]. The overall energy intake and macronutrient profile accords with one previous report in obese pregnancy [38]. Because of the time required to rigorously assess diet using the 24 hr recall method which, according to the process evaluation is likely to have influenced recruitment and compliance, a short food frequency questionnaire (5–10 minutes) was evaluated for use in the subsequent RCT.

The few studies that have attempted to measure changes in PA in intervention trials in pregnancy have generally relied on self-report, and results have been equivocal [38,46-49]. Accelerometry, the standard method of objective assessment used previously in observational studies in pregnancy [48], has, to our knowledge, only been employed in one relevant RCT, the FitFor2 study, a supervised exercise intervention in 121 overweight and obese women [50]. Consistent with Fitfor2, we found no effect of the intervention on PA using the Actigraph accelerometer, concurring with the reported absence of change in barriers to PA. The failure of accelerometry to mirror the increase in self-reported walking in the intervention group could reflect insufficient intensity of this activity, but also reporting bias [51] which is common in the reporting of low intensity activities, such as those frequently undertaken by pregnant women [52].

As reported elsewhere, compliance with accelerometry in pregnancy was an issue [53,54]. Nonetheless, 60% of obese pregnant women providing baseline accelerometry data met the current guidelines for PA in pregnancy (i.e. > 30 minutes of MVPA per day). A similar level of activity has been observed in pregnant women (all BMIs) [54] and overweight and obese non-pregnant adults [55], but not previously amongst obese pregnant women. Levels of PA were similar to those we found previously among overweight and obese women [53], but substantially higher than those reported for non-pregnant women in the UK [56]. There is no consensus on change of MVPA over pregnancy [53,57-59]. In this study of obese women both groups reduced the level of objectively measured MVPA as pregnancy progressed.

This assessment has highlighted a critical need to evaluate PA behaviour objectively. We may otherwise have erroneously concluded in the following RCT that increased PA does not affect clinically relevant outcomes. Despite showing no increase in PA, we have not recommended that the RCT focuses on diet only [4], but rather that women continue to be encouraged to adhere to PA recommended in clinical guidelines.

Although there were no changes in attitudinal outcomes, women were generally positive about the recommended dietary and physical activity behaviours despite perceived barriers to change. Attitudinal data relating to diet were comparable to a population sample of pregnant women [12]. The intervention did not achieve any reduction in perceived barriers, but despite this, important dietary changes were achieved which may infer low levels of self efficacy. However, barriers to increasing PA appeared too great to overcome, possibly reflecting increased physical discomfort with gestation, as indicated by the EQ-5D questionnaire.

The relationship between mental health, diet and PA in obese pregnancies warrants further investigation in the RCT in view of the high prevalence of depressive symptoms (EPDS score > 12). Another report has also found no effect of a complex behavioural intervention in obese women on these symptoms [60].

In terms of *context*, the process evaluation recruited women in urban hospitals serving regions with areas of high socio-economic deprivation. Obesity rates are higher amongst women with lower socio-economic status, fewer qualifications [61] and amongst particular ethnic groups, particularly black African and black Caribbean [62]. It was important therefore not only to explore if recruitment was feasible but also whether the intervention was acceptable to the women recruited. Prominent media coverage about obesity raised the possibility that women in the control group might proactively address diet and physical activity, and some interview data supported this, but the evaluation suggested that awareness through the media alone was not adequate to achieve sufficient behavioural change.

In relation to *reach*, just over one third of eligible women agreed to participate. Similarly low recruitment rates are consistent with other intervention studies, particularly in populations with lower uptake of health care. In one previous relevant study of lifestyle advice in non-obese pregnant women, recruitment was slower than expected and low attendance at group exercise sessions and participant concern about burdensome data collection contributed to dropout [63]. However, perceived advantages to participation such as extra clinical tests and continuity of care from research midwives supported study uptake and continuation. Given the continued rise in obesity in the adult population in England [61], approximately 1:5 pregnant women would be eligible for inclusion. Recruitment of the numbers needed to be approached (4100) for the full trial is therefore unlikely to be affected by a shortage of eligible women. Overall, the wide social and ethnic diversity amongst participants was similar in participants and those who declined, indicating that the intervention would be unlikely to increase health inequalities by attracting more educated and higher income participants [64]. Importantly, although obese pregnant women, once recruited, were generally willing to attend group sessions, practicalities often interfered with regular attendance, thereby influencing *dose*. However, the sessions did not appeal to all women. Some appreciated finding common issues with other group members, others preferred one to one contact. Evidence for health improvement interventions in group settings is varied [65] and this study adds to the recognition that a ‘one size fits all’ approach may not be effective [64], and that flexibility is key to retention. *Fidelity* was good with consistently high level provision of SMART goals by HTs, which were viewed as a positive achievement, particularly since poor adherence to goal setting has been associated with moderate attendance amongst pregnant women [66]. The high *acceptability* of the participant handbook and pedometer re-enforced the theoretical approach [16], and women also responded well to motivational techniques, but physical issues presented barriers to PA. The information provided was valued, including increasing awareness of safe PA in pregnancy, and seen to have important educational benefit. Several components of the intervention therefore appeared beneficial and were well received by women. The intervention is relatively intensive and presents costs for providers, and whilst a full assessment of cost and benefit was not conducted in this pilot, steps taken during the pre-clinical development phase (using HTs rather than clinicians to deliver the intervention, adopting local group-based approach) helped keep the overall costs of the intervention low, recognising that if beneficial, it should also be affordable to health providers and to women. There was also suggestion that the intervention may extend to peers and family, and some women aspired towards better fitness following birth. This study has reinforced earlier reports suggesting that rapport between study staff and participants, interviews requiring short time commitment, and participants' perception of the study as informative are all important recruitment and retention factors [67]. Formal evaluation of the reasons for the high refusal rate was not permissible due to ethical constraints, but the time commitment was frequently commented upon by the recruitment staff, as well as lack of appreciation of the health consequences of obesity in pregnancy.

In summary, this study has emphasised the value of a pilot trial to assess anticipated behaviour change. Although seldom attempted by others, we have also highlighted the importance of process evaluation in a complex intervention of diet and physical activity for pregnant women. The pilot trial demonstrated reductions in glycaemic load and in the proportion of energy derived from saturated fat are achievable in obese pregnant women without GDM. The process evaluation identified that dietary advice and education were well received, and confirmed that PA change is more problematic to achieve, although it remains important to consistently measure and support PA using technologies acceptable to women. The process evaluation also helped explain issues arising in relation to uptake, dose, fidelity and retention which informed the feasibility of the full trial.

As a consequence of this study, several modifications to improve compliance and fidelity have now been implemented in the protocol for the main trial. As well as process evaluation, HT feedback highlighted potential barriers to fidelity of the intervention and informed protocol modifications for the RCT. Flexibility has been increased regarding the timing and delivery of the sessions, and goal setting can be undertaken by telephone or email. It is recommended that women should receive at least 5/8 sessions. The two extra visits required for objective assessment of PA and accurate evaluation of diet have been omitted in all but two sites (as planned) and dietary assessment reduced to a validated FFQ. The RPAQ includes domestic and childcare activities, considered appropriate for pregnant women [68], but following feedback has been replaced by the shorter and more relevant IPAQ [69]. Maintenance of dietary and PA behaviour change in the participant and her family is being formally evaluated at 6 months and 3 years postpartum. To minimise loss to follow-up and attendance at these appointments, strategies which have been put in place include regular newsletters and sending greetings cards on special occasions such as the child’s birthday.

## Conclusions

Assumptions should not be made that interventions in obese pregnant women necessarily change behaviour. We recommend that a pilot trial such as that described here, which has demonstrated evidence for anticipated change in behaviour, is a necessary prelude to any RCT of a complex intervention of diet and physical activity designed to improve pregnancy outcome in obese women. Without prior evidence for change in behaviour in the anticipated direction, pursuit of a large and costly trial would be futile. Similarly, we have demonstrated the value of early process evaluation, which can lead to important refinements in protocol to improve feasibility and compliance in the definitive trial.

## Competing interests

The authors declare that they have no competing interests.

## Authors’ contributions

LP, ALB, RB, HC, NK, TK, TABS, JS, LMH EO-N and JW conceived the study, participated in its design and coordination and helped to draft the manuscript. SB, HC, KC, CH, LH, HNE and PTS performed the analyses. SB, HC, LMH, LH, NK, TK, SMN, EO-N, LP, NS, SCR, JW, TABS, JS helped to draft the manuscript. All authors read and approved the final manuscript.

## Pre-publication history

The pre-publication history for this paper can be accessed here:

http://www.biomedcentral.com/1471-2393/13/148/prepub

## Supplementary Material

Additional file 1: Table S1 Summary of session content; **Table S2**, Process intervention findings; **Table S3**, Structured interview sample characteristics; **Table S4**, Evaluation of intervention components. Click here for file
