# Supplementary material for: Developing a complex intervention for diet and activity behaviour change in obese pregnant women (the UPBEAT trial); assessment of behavioural change and process evaluation in a pilot randomised controlled trial
Source: BMC Pregnancy Childbirth. 2013 Jul 15;13:148. doi: 10.1186/1471-2393-13-148 (PMC3718630; doi:10.1186/1471-2393-13-148)
Supplement: Additional file 1: Table S1 — Summary of session content; Table S2, Process intervention findings; Table S3, Structured interview sample characteristics; Table S4, Evaluation of intervention components. [file 1471-2393-13-148-S1.doc]

**Additional Table 1:** Summary of session content

| Session and main topics | Content |
| --- | --- |
| **Introductory 1-1 session** Introduction to the programme | Overview of programme structure, content and participant materials  Participant expectations of taking part and what they hope to gain  Information regarding safe exercise in pregnancy  Participants given pedometer and information on how to use it  UPBEAT DVD and log book given |
| **Session 1**  Swapping soft drinks  First steps to physical activity | Introductions and group rules  Review of pedometer use  Information on soft drinks  Introduction to goal setting |
| **Session 2**  Reducing added sugar  Increasing everyday activity | Information on reducing added sugar  Information on SMART goal setting  Information on increasing everyday physical activity |
| **Session 3**  Swapping bread  Overcoming barriers to physical activity | Problem solving of barriers to being physically active  Information on choosing lower GI bread |
| **Session 4**  Swapping other starchy foods  Benefits of physical activity | Half-way review  Information on choosing lower GI rice, potatoes and other starchy foods  Information on benefits of physical activity  Review of own physical activity level  Discussion about the UPBEAT DVD |
| **Session 5**  Swapping snacks  Active leisure time | Information on choosing healthier snacks  Cravings vs. hunger  Discussion on leisure time physical activity and how to access services |
| **Session 6**  Swapping breakfast cereals  Local leisure services | Discussion on experiences of accessing local leisure services  Information on choosing lower GI breakfast cereals  Discussion on maintaining changes made to behaviour |
| **Session 7**  Choosing lower fat dairy products  Keeping active throughout the day | Review of dietary and activity topics covered so far, any benefits participants have experienced and accessing support after the sessions  Discussion on keeping active throughout the day  Information on choosing lower fat dairy products |
| **Session 8**  Choosing lower fat meat and meat products  Physical activity in late pregnancy | Information on choosing lower fat meat and meat products, and non-meat alternatives  Information on physical activity in late pregnancy  Maintaining behaviour changes |
| **Every session** | Review of goal attainment from previous week  Review of self-monitoring (log book) from previous week, including pedometer  Setting of dietary and activity SMART goals for coming week |
| **After the sessions**  (until 36 weeks) | Bi-weekly telephone support to discuss maintenance of behaviour changes and any further changes participants want to make |

**Additional Table 2:** Process intervention findings (context: interventions and controls)

**Context: Intervention and control participants**

It [Being part of the research] just makes you a bit more conscious of what you eat; even though you're pregnant and you might crave for all these different things, you think what impact it will have on you afterwards when, obviously when your baby’s born and you’re trying to get off the weight and it just makes you think of what you put in your mouth really. (ID 3, **control**, age 33, multiparous, BMI 44, Black African).

[Being part of the research has] reminded me a lot more, because I’m overweight, it could be that it was on the radio yesterday as well, I’m thinking about it a lot more…Obviously as part of the control group I’m not supposed to necessarily do anything specific and change anything but obviously I’m trying anyway. I don’t think I’d take part [in the research] if I regarded being overweight as perfectly alright and perfectly fine and I wasn’t interested in fixing the situation. (ID 12, **control**, age 35, multiparous, BMI 35, White British).

The midwife I've got through my GP isn't very friendly so I think it’s beneficial for me to come in and speak to [the research midwives] about the issues I have with the pregnancy and other stuff, because the one I have at my GP seems to be too busy to care, to listen to any of these things so it’s nice to come in and speak to them, it’s a bit reassuring I guess. (ID 1, **control**, age 26, nulliparous, BMI 37, black British).

I’ve really enjoyed it (blood tests, measurements, questionnaires) because it’s really reassuring, because you do a lot of the stuff with the midwives and you feel like if there was something wrong I’d be told about it. (ID 4, **intervention**, age 25, nulliparous, BMI 35, white British)

You feel more looked after. You feel like it’s more private care, more special than just the normal midwife care or the GP. Because they just want to get you in and out really quickly. Cos they have loads more people to see. But here you feel like you have the time to talk or say what you have to say. And ask any fears about anything, really, I think. (ID 17, **Intervention,** age 22, multiparous, BMI 37, mixed ethnicity).

**Additional Table 3:** Structured Interview sample characteristics

|  | **n** | **Interview Sample**  **Mean (SD)** | **Pilot trial population (n=183)** |
| --- | --- | --- | --- |
| **Control** | 12 |  |  |
| **Intervention** | 9 |  |  |
| **Ethnicity** |  |  |  |
| White | 8 | 38% | 56% |
| Black | 12 | 57% | 38% |
| Asian | 0 | 0% | 2% |
| Other | 1 | 5% | 4% |
|  |  |  |  |
| **Age** |  | 29.6 (4.9) | 30.5 (5.4) |
|  |  |  |  |
| **Parity** |  |  |  |
| Nulliparous | 9 | 43% | 44% |
| Multiparous | 12 | 57% | 56% |
|  |  |  |  |
| **Gestation at interview** (weeks) |  | 29 weeks | Not interviewed |
| **BMI at recruitment (kg/m^2^)** |  | 37.6 (4.6) | 35.6 (5.1) |

**Additional Table 4.** Evaluation of intervention components

**Dose received and acceptability of study components: Intervention group**

*Expectations of the intervention*

I thought it was going to be healthy eating and exercising. I thought it was going to be like how they tell you in the news,that we have to eat better. Or what you hear media- wise. But it’s more in-depth and more suitable to how you are, basically. It’s more fitted to how you are. Instead of every thousand people. It’s just for you. It’s more suitable that way, I feel. (ID 17, **Intervention,** age 22, multiparous, BMI 37, mixed ethnicity).

The groups that I went to, there was only … two of us at maximum. I think there were supposed to be four. (ID 4, **intervention**, age 25, nulliparous, BMI 35, white British).

*Access to intervention*

I couldn’t come to all the sessions, I think it started at 3 and school finishes at 3:15 so it’s a bit difficult to try and get [to the hospital] for that time. (ID 2, **intervention,** age 27, multiparous, BMI 34, black African).

The only thing I was worrying about was being able to commit every week. But I think there was once that I couldn’t do it and [HT] e-mailed and she phoned so I didn’t miss out on anything. (ID 8, **intervention**, age 29, BMI 43, white British).

I also found it quite hard saying ‘Oh, I need eight Thursday afternoons off work’, and I just felt like I was taking advantage of them by taking extra time off work. (ID 4, **intervention**, age 25, nulliparous, BMI 35, white British).

*Affect on mood*

… I just felt quite … quite bad, and I felt that … that I wasn’t doing good … I wasn’t doing what was good for my baby … by not being healthy and fit and … and all of that, I felt like I was doing something wrong, so … I don’t know…you’re on a diet of guilt, you know, you should be eating this because otherwise you’re doing badly. (ID 4, **intervention**, age 25, nulliparous, BMI 35, White British).

…because you’re on the [research] project, you feel good when you do something good. And when you do something bad, you feel bad. Because you feel like you’re letting yourself down. (ID 17, **Intervention,** age 22, multiparous, BMI 37, mixed ethnicity).

*Handbook and dietary change*

Instead of the basmati rice, I’d had the normal long grain rice, and instead of mashed potato, you can have sweet potatoes, it’s just really silly things you didn’t know, you thought you were eating healthily and you weren’t, so changing that, swapping… The benefits are you’re definitely not gaining that much weight, which is a plus. All the women will like that bit of it, so it kept you going. (ID 13, **intervention**, age 36, multiparous, BMI 32, black African).

I’ve always bought wholemeal bread but because we were encouraged to buy seedy bread, I am still buying it and I think I’ll continue to buy it. (ID 11, **intervention**, age 35, multiparous, BMI 41, black African).

I don’t know how to say it, [the handbook] was more for people who didn’t really have … good knowledge with food, or cooking or eating well, do you know what I mean? It’s just like … I eat healthy… maybe too many desserts or cakes or sweets or whatever, but I do know *how to* eat healthy, but [the handbook] was more aimed at people who don’t know, who are just eating for the sake of eating. (ID 4, **intervention**, age 25, nulliparous, BMI 35, white British).

*Physical activity*

The logbook was fantastic with the pedometer. It was so motivating. We were aiming for 10,000 steps a day so every day I was doing extra trying to get to that and that really motivated me and the family, all the kids were behind me. (ID 13, **intervention**, age 36, multiparous, BMI 32, black African).

If I didn’t leave home at all [one day], I would have like 1000 steps and I’m like “Oh my God, that’s really [low]...” it sort of motivated me to try and do something about it the next day. …if I hadn’t done this [research], I suppose I’d still be in that mindset, that, “Oh, I am pregnant, I’m not allowed to do anything” whereas now, because of having looked at my step count, I am very aware that I have to stay active and when I don’t, it does bug me. (ID 11, **intervention**, age 35, multiparous, BMI 41, black African)

I developed a condition called SPD, it was my pelvis which becomes really unbearable and very painful to walk…but obviously after this baby comes I am going to make a conscious effort to do a lot more. And obviously I’ll not have the problems with my pelvis as well which will be a great help. (ID 8, **intervention,** age 29, nulliparous, BMI 43, white English).

I want to start on my diet after my baby’s born. More healthy cooking and stuff. And once or twice a week, swimming and stuff like that. It makes you feel positive about yourself to do more. So afterwards you feel, okay, if I can do this while I’m pregnant, I can do 100 times more when I’m not. So I think it’s a motivation thing. It makes you think about, basically, it makes you think about your health during your pregnancy. But it makes you think afterwards, as well, so if I can take this much care when I’m pregnant, I can do a lot more afterwards. (ID 17, **Intervention,** age 22, multiparous, BMI 37, mixed ethnicity).
